# Key informant perspectives on policy- and service-level challenges and opportunities for delivering integrated sexual and reproductive health and HIV care in South Africa

**DOI:** 10.1186/1472-6963-12-48

**Published:** 2012-02-27

**Authors:** Jennifer A Smit, Kathryn Church, Cecilia Milford, Abigail D Harrison, Mags E Beksinska

**Affiliations:** 1 MatCH (Maternal, Adolescent and Child Health), Department of Obstetrics and Gynaecology, University of the Witwatersrand, Durban, South Africa; 2 Department of Global Health and Development, Faculty of Public Health and Policy, London School of Hygiene and Tropical Medicine, London, UK; 3 Population Studies and Training Center, Brown University, Rhode Island, USA

**Keywords:** HIV, Family planning, Reproductive health, Sexual health, Integrated delivery of healthcare, South Africa

## Abstract

**Background:**

Integration of sexual and reproductive health (SRH) and HIV services is a policy priority, both globally and in South Africa. Recent studies examining SRH/HIV integration in South Africa have focused primarily on the SRH needs of HIV patients, and less on the policy and service-delivery environment in which these programs operate. To fill this gap we undertook a qualitative study to elicit the views of key informants on policy-and service-level challenges and opportunities for improving integrated SRH and HIV care in South Africa. This study comprised formative research for the development of an integrated service delivery model in KwaZulu-Natal (KZN) Province.

**Methods:**

Semi-structured in-depth interviews were conducted with 21 expert key informants from the South African Department of Health, and local and international NGOs and universities. Thematic codes were generated from a subset of the transcripts, and these were modified, refined and organized during coding and analysis.

**Results:**

While there was consensus among key informants on the need for more integrated systems of SRH and HIV care in South Africa, a range of inter-related systems factors at policy and service-delivery levels were identified as challenges to delivering integrated care. At the policy level these included vertical programming, lack of policy guidance on integrated care, under-funding of SRH, program territorialism, and weak referral systems; at the service level, factors included high client load, staff shortages and insufficient training and skills in SRH, resistance to change, and inadequate monitoring systems related to integration. Informants had varying views on the best way to achieve integration: while some favored a one-stop shop approach, others preferred retaining sub-specialisms while strengthening referral systems. The introduction of task-shifting policies and decentralization of HIV treatment to primary care provide opportunities for integrating services.

**Conclusion:**

Now that HIV treatment programs have been scaled up, actions are needed at both policy and service-delivery levels to develop an integrated approach to the provision of SRH and HIV services in South Africa. Concurrent national policies to deliver HIV treatment within a primary care context can be used to promote more integrated approaches.

## Background

Integrating sexual and reproductive health (SRH) and HIV services is an important health policy concern, especially in high-HIV prevalence settings in sub-Saharan Africa [1,2]. A history of verticalized programming in the region has resulted in SRH and HIV services commonly being delivered in separate or semi-specialized facilities and units [3]. The disjuncture has become even more marked as HIV services have been rapidly scaled-up in high-prevalence settings, including HIV counseling and testing (HCT), prevention of mother-to-child transmission (PMTCT) and HIV treatment services [4]. Further, while SRH services, in particular family planning (FP) and maternal and child health (MCH), may have been considered integral components of a generalist primary health care (PHC) structure, treatment-focused HIV services have often been delivered within more specialist units in tertiary health facilities, or by health workers with a sub-specialism in HIV [5,6].

There have been calls for the implementation of integrated health services to address the fragmentation of both policies and programs in the two spheres of SRH and HIV [3,7]. Following the International Conference on Population and Development (ICPD) (1994), service integration in the field of SRH has been interpreted as offering a range of services that could meet several health needs simultaneously, usually at the same time, same facility, and with the same provider [8]. The approach is inherently complex, however, since multiple service configurations can operate in different settings, and challenges in implementation were identified soon after the ICPD conference [9]. Recently policy attention has shifted to promoting *linkages *between services, which may involve improved referral services, in addition to policy and program coordination [10]. Since most HIV infections are sexually transmitted or associated with pregnancy, childbirth and breast-feeding, there is a strong and logical rationale to integrate care. Some suggest that integrated services promote access to care, reduce costs and may be less stigmatizing for those accessing dedicated HIV services [11,12].

Attention to service integration has been manifest in South Africa, which has the greatest number of HIV-infected people in the world [13]. While a PHC approach was adopted in 1994 to ensure the delivery of equitable, accessible, comprehensive and integrated healthcare [14], there were challenges in delivery and scale-up. These included difficulties in ensuring an integrated approach within decentralized health services that were supported by vertical national health programs and subject to widespread human resource shortages and skills [15,16]. Moreover, the rapid implementation of HIV services, though essential to address the epidemic, may have paradoxically reinstated the very same vertical, fragmented approach to health that the country strived to eliminate in the 1990s [17-19]. Studies have documented ongoing missed opportunities within South African health services to address both the HIV prevention and treatment needs of FP and MCH clients [20,21], and the SRH needs of a growing number of HIV counseling and testing (HCT) and HIV treatment clients [6,22].

There is therefore a growing imperative to develop and implement innovative policies and service-delivery modalities that enable SRH and HIV care to be addressed in a genuinely integrated fashion. This study aims to achieve a better understanding of the current approach to service delivery, potential challenges to integrating care, and current opportunities for moving the agenda forward, to help inform such processes. Since much of the existing research in South Africa exploring these themes was conducted prior to the mass scale-up of HIV treatment programs [15,16,20,21] and more recent studies focus primarily on the SRH needs of HIV patients, and less on the policy and service-delivery environment in which programs are operating [6,22,23], we aim to fill an important gap in the literature.

This qualitative study forms part of a larger research program that aimed to develop and evaluate a district-based model of integrated SRH and HIV services in KwaZulu-Natal (KZN) Province. Data from these key informant interviews (KIIs) facilitated the design of the integration intervention, tailored to specific sites and situations. This integration model is currently being evaluated through a prospective pre-/post-test design using exit interviews, as well as a process evaluation through further qualitative research.

## Methods

A qualitative approach was chosen to provide an in-depth understanding of specific contexts considered essential to developing an innovative model for integrating SRH and HIV services. Twenty-one key informants were interviewed (4 male, 17 female), including policy-makers, program managers, and academics working in the health policy and SRH/HIV field (see Table 1). Individuals at a local, national and international level who had knowledge and experience of integrating SRH and HIV services in South Africa were selected purposively to ensure that appropriate informants would provide rich study data [24]. In addition, snowball sampling, a strategy whereby experts help identify other information-rich cases, was used. Sample size was determined by the data obtained, and we sampled to redundancy [24]. The final sample comprised 12 Department of Health (DoH) employees (national, KZN provincial and district levels), five academics (from South African and international institutions), and four representatives of South African non-governmental organizations (NGOs) working in SRH and HIV. All provided written informed consent. Four people from the National DoH declined to participate in the study (either due to perceived lack of expertise or time constraints).

**Table 1:** Background characteristics of key informants

	n (21)
**Sector:**	
Department of Health	12
NGO	4
Academic (International)	2
Academic (South African)	3

**Sex:**	
Male	4
Female	17

**Age (years):**	Range: 31-68
	Median: 47

**Number of years working in field:**	Range: 3-40
	Median: 10.5

**Figure 1:**
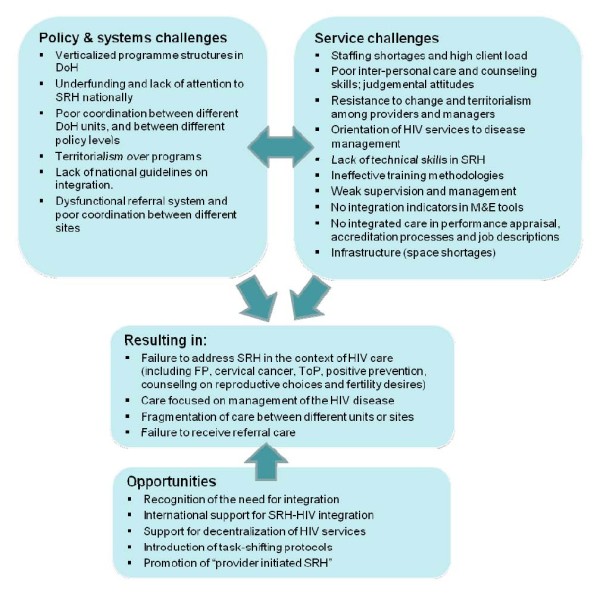
**Challenges & opportunities to delivering integrated SRH and HIV care**.

A semi-structured interview guide explored current SRH and HIV policy and service availability and integration issues (including understanding of integrated care, challenges and benefits to integration, and ideal service-delivery models), both in KZN and in South Africa more broadly. Interviews were face-to-face or telephonic, audio-recorded and transcribed. Transcripts were entered into NVivo 8 to facilitate data analysis. An initial list of thematic codes generated from a subset of the transcripts was developed by two independent researchers to ensure reliability. These codes were then modified and refined through an inductive analytical approach, and results organized according to key themes that emerged from the data (as summarized in Figure 1).

The study was approved by the Human Research Ethics Committee of the University of the Witwatersrand (#M080624).

## Results

While informants were drawn from NGO, academic and government sectors, data are presented here according to two key levels of influence on integrated SRH-HIV care provision in South Africa: the policy level (primarily national, but also factors influencing coordination between national, provincial and local government), and the service-delivery level (from experience either within KZN or other provinces) (see summary in Figure 1). Differences between views of informants from different levels of government were not found. Opportunities to advance an integrated care agenda in South Africa are discussed.

### Policy & systems challenges to integrated SRH-HIV care

The lack of national policy guidance on integrated care was highlighted by many informants as an impediment to integration. While some mentioned previous attempts to include SRH within HIV policies, it was clear that most guidelines, in particular clinical service guidelines, remain specialized. The separation of SRH and HIV services under different directorates within both the national and provincial DoH was also considered problematic. Coordinated planning was seen to be inhibited by program territorialism and budgetary concerns:

People have been owning certain projects and they're resistant now, ... because they had this project running with their own resources and when we integrate now, it says we're going to share our resources.(DoH)

The recent prioritization of funding for HIV was seen to have contributed to verticalization of health care programming and there was consensus that SRH services across South Africa had been sidelined:

...[FP] services are really struggling [...] there's been such a push to [...] get these HIV and AIDS services up, and a lot of the dynamic people I know at district level have gone over into HIV services. (Academic)

Many informants thus emphasized the need for improved coordination and collaboration between different levels, tiers and authorities of the public health sector to bridge the separate *"silos" *of SRH and HIV programming, as well as the need for joint responsibility and accountability of outcomes.

Verticalized programming across national, provincial and local health directorates was also seen to have contributed to care fragmentation across different tiers of the service hierarchy, resulting in certain services being available only within PHC (e.g. FP), and others only at hospitals (e.g. cervical cancer treatment). For some services, such as STI treatment, it was unclear where they belonged, resulting in patients being cross-referred multiple times. Some interventions, including HCT or CD4 testing, may also need to be repeated on multiple occasions in different sites due to differing national and provincial program requirements. A picture thus emerged of clients, in particular HIV treatment clients being routinely sent *"from one service point to another" (DoH)*.

Referral systems were also considered weak due to a range of factors, including a lack of clear policies on what can and should be addressed in different SRH or HIV units; insufficient client counseling on the need for referral services; distance to sites and client transport costs; and poor communication between referral sites. The need for improving coordination between facilities within KZN province and elsewhere was highlighted, including facilitating transfer of patient records:

...sometimes integration can mean that when you refer somebody, you do it sort of properly, you have a relationship with that other facility, that you provide notes that they will know, you have some feedback. (Academic)

### Service level challenges to integrated SRH care

At the service level, it was reported that SRH services for people living with HIV/AIDS (PLWHA) are often neglected, both in KZN and elsewhere in South Africa. The need to promote contraceptive services as an integral component of national HIV care and treatment programs was highlighted by all informants, and access to other SRH services for PLWHA was also considered poor, including counselling on reproductive choices, positive prevention counselling, cervical cancer screening, abortion and assisted conception services:

Nobody's addressing the reproductive health of a patient at the ARV clinic. I would like them to stand up and talk about it yes, if anybody would like to have a child or would like to do this, or if you fallen pregnant by mistake, this what you can do, this is the procedure...(DoH)

Staffing shortages and high client load were the most commonly discussed service-related factors influencing integrated care. Many services were considered to be operating at *"their bare minimum"*, resulting in long queues and waiting times:

The bottom line is that you don't have enough health workers. Then, if you want to add services and you want to integrate them and link them, you need more people... (Academic)

It was felt unrealistic to expect providers to explore multiple health needs within a typical consultation timeframe (six to seven minutes). Staff burnout, sickness, absenteeism, attrition, administrative duties, frequent rotation and training were also mentioned as contributing factors. It was noted that current organizational systems (i.e. requiring clients to come in the morning without appointments) push providers to deal quickly with patients, allowing them to do paperwork in the afternoons. However, it was felt that a basic level of integration was achievable despite staff challenges. As one informant noted, it may *"only add seconds"(Academic) *to a consultation to discuss potential needs such as FP.

Negative attitudes of providers towards integration were also highlighted, with some purportedly *"completely opposed to integrating or even [...] referring patients for some services"(NGO)*. Several informants, primarily from NGO or academic sectors, felt that the failure to counsel PLWHA on fertility choices stemmed partly from negative attitudes towards pregnancy in HIV-positive women. Provider inhibitions in counseling on sexual matters were mentioned, and several informants proposed values clarification workshops to address judgmental attitudes.

Resistance to change among providers and service managers was raised as an additional barrier to integration, either stemming from fear of increased workloads and responsibilities, or due to territorialism at the service level. Informants talked of more specialized providers, such as HCT counselors, *"trying to hold on to their jobs"(NGO)*. However, some felt that attitudes are changing and that integrated approaches would be empowering for providers and lead to more effective collaboration:

I think with time people will realize that it's for the good of the patient, it will also improve our collective spirit if we work together, plan together. [...] So that if you all work together it will also lessen the pressure and the volume of work on the healthcare worker. (DoH)

Lack of attention to the SRH needs of PLWHA was also linked to a wider failure of providers to *"look beyond the disease" *and treat patients holistically. Informants felt that HIV providers often focus on clinical management of HIV illness, partly due to the complexity of HIV disease management and burden of care, and they struggle to meet the multiple and chronic health needs of clients, including conditions directly related to HIV, such as tuberculosis: *"if they are on ART they will be treated as [an] HIV positive person, they will not be treated as a woman"(DoH) *and providers were reported to feel that *"we are overloaded, we can't be asking people about [...] contraception" (Academic)*. Providers in HIV services were also seen to lack the relevant technical skills and confidence required to deliver SRH services:

Providers just are not aware of what contraception should be used...providers have said to us, "we don't ask what contraception you are on, or whether you need it" because they don't know what the right answer is. (Academic)

New approaches to training were recommended, including integrated training courses for SRH and HIV, decentralized training at district level, appropriate pre-service training, and post-training mentorship. Suggested approaches to facilitate integrated care included screening and use of history-taking tools to support identification of multiple health and social needs.

Strengthening supervision and management at all levels was also emphasized as a critical need. One participant stressed *"if my head of department doesn't think it's important, nobody's going to look at it"(DoH)*. Informants proposed strategies to improve management, including: revising monitoring and evaluation systems, using indicators to measure integration activities, conducting advocacy with managers to promote their buy-in, revising job descriptions to include a broader scope of healthcare, offering financial incentives for providers to improve and broaden skills, and using performance evaluation and accreditation processes which include the delivery of integrated care.

Lastly, some informants felt that infrastructural changes within facilities were needed, to accommodate a broadened package of care, particularly in smaller facilities. For example, one informant questioned whether there was sufficient space in HIV clinics to routinely conduct cervical cancer screening.

### Looking ahead: Strategies and models for integrated SRH-HIV care

Despite the challenges, there was consensus from all respondents on the need to move forward and develop more integrated systems of care at local, provincial and national levels. Many informants advocated for the establishment of "one-stop shops" with a comprehensive range of services accessible within one facility. Their views varied, though, on how a precise model of integration would work within such a facility. Several thought that having one provider offer a range of SRH and HIV services would be ideal ("*full integration" *or *"provider-level integration"*), overcoming problematic referral processes and reducing the need for clients to queue multiple times. But it was acknowledged that having one provider do everything may not be realistic or achievable, in particular within HIV care:

If they are really overwhelmed with testing and CD4 cell counts, and dishing out drugs, then I think it's utterly naïve to think that same overworked set of staff can be nudged into talking about childcare and family planning. (Academic)

Further, the potential for losing critical medical competencies through a more generalist approach was considered a risk with this model: *"how many of us can claim to be an expert on everything?"(DoH)*. Several informants felt the need to maintain sub-specialist providers, and proposed a team-based approach to comprehensive care, particularly in larger facilities. The concept of *"partial" *or *"facility-level integration" *was proposed, implying internal referral to access SRH services within the same facility:

...integration isn't about doing everything together necessarily. It's about who provides what, and what are the linkages between the two. And so things can be integrated in more than one way. They don't have to all be in the same room, [...] I would rather see somebody doing family planning everyday and doing abortions everyday and not having to do the whole [...] thing, but I would like to see them in the same clinic. (Academic)

In both of these models, informants felt that the onus remains with the health worker to identify and/or proactively raise potential health issues. In this context, the concept of "provider-initiated SRH" was raised:

...now that we are coming with strategies like provider-initiated [HCT], maybe we need to [...] say we have provider-initiated family planning, you know? [...] we need to reach that stage, whereby each and every health worker is talking about the language. [...] I think it should be integrated in each and every section within the facility... (DoH)

Several current health systems strategies in the reorganization of South African healthcare were raised as potential opportunities for integration. Firstly, it was felt that task-shifting would free up the time of nurses and doctors to deliver more comprehensive clinical care. For example, counselors could deliver health talks on SRH issues at HIV clinics; or booking clerks could facilitate client screening. Secondly, the decentralization of HIV treatment to PHC would facilitate integration with SRH services, since the latter are typically available at that level. Lastly, ongoing efforts to coordinate the work of different health departments were reported, including the development of joint policies (for example through the *HIV and AIDS and STI Strategic Plan for South Africa, 2007-2011) *and integrated patient management strategies like the *Integrated Management of Adolescent and Adult Illnesses (IMAI) *or *Basic Ante-Natal Care (BANC)*, which address both SRH and HIV issues through clinical algorithms which diagnose and address multiple health care needs.

## Discussion

### Limitations

Findings were based on opinions and experiences of key informants, rather than on empirical data from clinics. However, high levels of agreement among the different key informants suggest reliability of the findings. The views and opinions of the key informants may not be representative of all experts in KZN or in South Africa more broadly. International factors which may play a role in health policy at that national level were not addressed in this study, and international informants spoke specifically to challenges from their experience working in South Africa.

### Summary of findings and implications

Despite the progressive approach to the delivery of comprehensive and integrated health services adopted in South Africa in the mid-1990s, our findings demonstrate the persistence of vertically configured services, with a narrow focus on HIV treatment as the priority for health service delivery, and insufficient attention to and resources for SRH services, including for PLWHA. While other South African studies on SRH-HIV integration have documented coverage gaps in SRH and HIV services [6,25], this study has clearly highlighted how verticalized health programming, especially for HIV, contributes to a failing in integrated care provision. There was consensus among our informants regardless of sector or level of government from which they were drawn, on the need for more integrated systems of SRH and HIV care.

Despite the long-standing global recognition of the need for the integration of basic health and SRH services with HIV services [1], our data provide a perspective on inter-related systems factors at policy and service-delivery inhibiting the delivery of integrated care. Separate policies, guidelines, ministerial directorates, under-funding of SRH, program territorialism, weak management systems, vertical training programs, lack of monitoring and evaluation systems, and ineffective referral systems were considered critical barriers to integration. Providers therefore understandably struggle to cope with multiple health programming and service delivery requirements. The capacity of already overburdened staff to address diverse and broad client health needs also remains a concern given the human resource crisis in the South African health sector [18]. While some solutions were proposed, e.g. task-shifting, team work, or decentralization of HIV treatment, the complexity of managing HIV in this high prevalence setting will continue to impact on health workers, limiting their capacity to address their clients' more holistic healthcare needs. Policy directives mandating the delivery of health care in an integrated fashion are needed to normalize integration as a requirement, rather than an optional extra. Support, guidance and training to facilitate the integration of services are necessary to enable providers and managers to understand the need for and provide integrated SRH care, including its potential to prevent onward HIV transmission by addressing unmet FP needs [26].

## Conclusion

The enthusiasm for integration among local and national policy-makers and program managers was undisputed and although informants' opinions diverged on the best model to achieve integrated care, there was agreement that concerted action is needed. An incremental approach to service integration may be most practical and feasible given the challenges raised. The proposal to integrate FP services into HIV clinics through "*provider-initiated family planning" *was noteworthy, and could be a good place to start given that contraceptive services were seen as a critically neglected area. Maximizing opportunities to provide FP at routine HIV visits could achieve a degree of service integration without placing a huge additional burden on the health system. Development of policy and guidelines for "provider-initiated SRH" in HIV services could support more comprehensive, more integrated care for PLWHA, in both South Africa and other high HIV prevalence settings. Actions should also be considered to address HIV more comprehensively in SRH settings, e.g. by ensuring the implementation of provider-initiated HCT in those settings.

## Abbreviations

ARV: Anti-retroviral; DoH: Department of Health (South Africa); FP: Family planning; HCT: HIV counseling and testing; ICPD: International Conference on Population and Development; KIIs: Key informant interviews; KZN: KwaZulu-Natal (Province); MCH: Maternal and child health; NGO: Non-governmental organization; PHC: Primary health care; PLWHA: People living with HIV/AIDS; PMTCT: Prevention of mother-to-child transmission (of HIV); SRH: Sexual and reproductive health; STI: Sexually transmitted infection.

## Competing interests

The authors declare that they have no competing interests.

## Authors' contributions

JAS, CM, ADH and MEB contributed to the conception and design of the study. JAS and CM oversaw data collection. CM, KC and MEB conducted interviews with key informants. CM and KC undertook data coding and data analysis with oversight from JAS, MEB. JAS and KC drafted the manuscript, which was commented on and revised by all authors. JAS, KC, CM and MEB finalized the manuscript. All authors read and approved the final manuscript.

## Pre-publication history

The pre-publication history for this paper can be accessed here:

http://www.biomedcentral.com/1472-6963/12/48/prepub
